# Solution structure and tandem DNA recognition of the C-terminal effector domain of PmrA from *Klebsiella pneumoniae*

**DOI:** 10.1093/nar/gkt1345

**Published:** 2013-12-25

**Authors:** Yuan-Chao Lou, Iren Wang, M. Rajasekaran, Yi-Fen Kao, Meng-Ru Ho, Shang-Te Danny Hsu, Shan-Ho Chou, Shih-Hsiung Wu, Chinpan Chen

**Affiliations:** ^1^Institute of Biomedical Sciences, ^2^Institute of Biological Chemistry, Academia Sinica, Taipei 115, ^3^Institute of Biochemistry and ^4^Agricultural Biotechnology Center, National Chung Hsing University, Taichung 40227, Taiwan, Republic of China

## Abstract

*Klebsiella pneumoniae* PmrA is a polymyxin-resistance-associated response regulator. The C-terminal effector/DNA-binding domain of PmrA (PmrA_C_) recognizes tandem imperfect repeat sequences on the promoters of genes to induce antimicrobial peptide resistance after phosphorylation and dimerization of its N-terminal receiver domain (PmrA_N_). However, structural information concerning how phosphorylation of the response regulator enhances DNA recognition remains elusive. To gain insights, we determined the nuclear magnetic resonance solution structure of PmrA_C_ and characterized the interactions between PmrA_C_ or BeF_3_^−^-activated full-length PmrA (PmrA_F_) and two DNA sequences from the *pbgP* promoter of *K. pneumoniae*. We showed that PmrA_C_ binds to the PmrA box, which was verified to contain two half-sites, 5′-CTTAAT-3′ and 5′-CCTAAG-3′, in a head-to-tail fashion with much stronger affinity to the first than the second site without cooperativity. The structural basis for the PmrA_C_–DNA complex was investigated using HADDOCK docking and confirmed by paramagnetic relaxation enhancement. Unlike PmrA_C_, PmrA_F_ recognizes the two sites simultaneously and specifically. In the PmrA_F_–DNA complex, PmrA_N_ may maintain an activated homodimeric conformation analogous to that in the free form and the interactions between two PmrA_C_ molecules aid in bending and binding of the DNA duplex for transcription activation.

## INTRODUCTION

Bacteria are highly adaptive organisms whose genomes harbor many genes and pathways for sensing and responding to environmental cues. The two-component system (TCS) is one of the major ways of coupling environmental stimuli to adaptive responses (1). A classical TCS typically consists of a transmembrane sensor histidine kinase (HK) and a cytoplasmic response regulator (RR) protein. After perceiving external stimuli by the sensor domain of the HK, a phosphoryl group on a highly conserved His residue of the HK is auto-generated and then transferred to the conserved Asp residue on its cognate RR protein to elicit adaptive responses. In pathogens, a number of TCSs are integrated and required for persistence in response to a wide range of stressors and environments and for providing virulence in host cells (2–4). TCSs are ubiquitous in bacteria but absent in mammals, so bacterial TCSs are potent targets for drug design, especially those that control virulence such as the PmrA/PmrB TCS (5).

Gram-negative bacteria resist being killed by antimicrobial peptides and avoid detection by host immune systems often by modifying lipopolysaccharide (LPS) in their outer membrane. The PmrA/PmrB TCS is the major regulator of genes for LPS modification in bacteria (6). The RR PmrA is activated when its cognate HK PmrB senses excess Fe^3+^, Al^3+^ and mild acidic environments (7–9). Also, at low Mg^2+^ concentration, PhoP/PhoQ, another virulence TCS, promotes the expression of a connector protein, PmrD (10), which can prevent the intrinsic dephosphorylation of phospho-PmrA and enhance the expression of PmrA-activated downstream genes (11). The genes activated by PmrA, including *pbgPE*, *cptA* and *ugd*, can encode enzymes to alter the composition of LPS, which increases the bacterial resistance to polymyxin B and other host-derived antimicrobial peptides (7,12) or allows for bacterial survival within macrophages (13). However, in addition to playing important roles in antimicrobial peptide resistance, PmrA was also found to limit *Salmonella* virulence by repressing the type-3 secretion system Spi/Ssa (14), which translocates effector proteins into and across the phagosomal membrane (15) and is necessary for bacterial survival within macrophages (16). Thus, the PmrA/PmrB TCS has two distinct contrary functions, one to modify LPS to increase bacterial resistance to antimicrobial peptides and another as an antivirulence factor. The antivirulence function of PmrA may limit the acute phase of *Salmonella* infection, thereby enhancing pathogen persistence in host tissues (14).

*Klebsiella pneumoniae* is a common cause of nosocomial bacterial infections causing pneumonia and urinary tract infections, especially in immuno-compromised patients (17). The increasing antibiotic resistance of *K. pneumoniae* emphasizes the importance of investigating how virulence and drug resistance persists through the PmrA/PmrB TCS. *Klebsiella pneumoniae* PmrA, which belongs to the OmpR/PhoB subfamily and has about 76% sequence identity to *Escherichia coli* and *Salmonella* PmrA (Supplementary Figure S1), is composed of an N-terminal receiver domain (PmrA_N_) and a C-terminal effector/DNA-binding domain (PmrA_C_). The activation of the RR in the OmpR/PhoB subfamily is initiated by phosphorylation of the Asp residue in the N-terminal receiver domain. This phosphorylation induces the formation of a head-to-head dimer in the N-terminal domain by a conserved α4 -β5-α5 interface, accompanied by the binding of a C-terminal effector/DNA-binding domain to the imperfect or perfect tandem repeat sequences on the promoters of target genes.

The structures of a large number of effector and receiver domains have been determined (18). However, the structures of the activated full-length OmpR/PhoB subfamily RR in free conformation or in complex with DNA are unknown. The structures of several inactive OmpR/PhoB subfamily members are all in a monomeric state with different domain arrangements (19–22). Some structures have extensive interfaces between N-terminal and C-terminal domains and others do not. The recognition helices of some inactive RRs are occluded, whereas those in other RRs are exposed. Moreover, the C-terminal DNA-binding domain of PhoB (PhoB_C_) binds to DNA as a head-to-tail dimer (23), whereas that of OmpR can contact DNA in head-to-tail or head-to-head orientations (24,25). These studies imply more divergent regulatory mechanisms in the OmpR/PhoB subfamily. Hence, structural studies of activated full-length RR and its DNA binding are crucial.

Previously, we determined the solution structure of *K. pneumoniae* PmrD and the X-ray structure of *K. pneumoniae* PmrA_N_ activated with the phosphoryl analog beryllofluoride (BeF_3_^-^) and characterized their interactions by NMR and several other biophysical methods (26,27). In this study, we focused on the C-terminal DNA-binding domain. We verified the PmrA_C_ binding sequences on the *pbgP* promoter of *K. pneumoniae* and analyzed their interactions with PmrA_C_ or BeF_3_^−^-activated full-length PmrA (PmrA_F_). We determined the solution structure of PmrA_C_ and the residues involved in DNA recognition. The structural basis of PmrA_C_–DNA interactions were modeled by HADDOCK and verified by nuclear magnetic resonance (NMR) paramagnetic resonance enhancement with spin-labeling of two thymines. Finally, we characterized the interaction between PmrA_F_ and box1 DNA by NMR and proposed the structural events for PmrA activation and DNA recognition.

## MATERIALS AND METHODS

### Preparation of recombinant proteins and oligonucleotides

The DNA fragments encoding full-length PmrA and PmrA_C_, the C-terminal fragment from residues Asn^121^ to Glu^223^ of PmrA, were cloned into a vector pET-29b(+) (Novagen) in *E. coli* strain BL21(DE3) with an extra Met residue at the N-terminus and an additional LEHHHHHH tag at the C-terminus for purification. The mutants were generated by the QuickChange site-directed mutagenesis protocol (Stratagene) and confirmed by DNA sequencing. For full-length PmrA, two residues were mutated (Trp^181^ to Gly and Ile^220^ to Asp) to improve solubility. The DNA-binding abilities of the wild-type and mutated full-length PmrA were similar (data not shown). For ^15^N/^13^C- or ^15^N/^13^C/^2^H-labeled protein samples, cells were grown in H_2_O or D_2_O containing M9 minimal medium supplemented with ^15^NH_4_Cl and ^13^C-glucose at 37°C. The cells were disrupted by use of an M-110 S microfluidizer (Microfluidics). Recombinant protein was purified by use of nickel-nitrilotriacetic acid affinity resin (Qiagen, Hilden, Germany). The purity of samples was checked with use of coomassie blue-stained sodium dodecyl sulphate (SDS) polyacrylamide gel and was >95%. Full-length PmrA was activated by BeF_3_^-^ as described previously (27).

The oligonucleotides used for NMR, fluorescence polarization and isothermal titration calorimetry were from MDBio Inc. (Taiwan). The denominations and sequences are box1 (5′-ATTTCTTAATATTATCCTAAGCAAG-3′), box1a (5′-AATTTCTTAATATTAT-3′), box1b (5′-ATTATCCTAAGCAAGG-3′), box2 (5′-TCATTTTAATTTCGTTTAAGTCCG-3′), box2a (5′-TCATTTTAATTT-3′) and box2b (5′-CGTTTAAGTCCG-3′), with the PmrA binding sites underlined. Double-stranded DNA was prepared by mixing an equal amount of two complementary oligonucleotides in 20 mM sodium phosphate and 30 mM NaCl at pH 6.0, heating to 95°C for 30 min and cooling slowly to room temperature. Double-stranded DNA for NMR was further purified on a Mono-Q 5/50 GL column (Amersham Biosciences) with elution by NaCl concentration gradient from 0.1 to 1 M. The concentrations of DNA and proteins were calculated by ultraviolet (UV) absorbance at 260 and 280 nm, respectively, with an ND-100 UV-Vis spectrophotometer (NanoDrop Technologies, Inc.).

### Fluorescence polarization measurements

For fluorescence polarization experiments, the oligonucleotides were labeled with 6-carboxyfluorescein (6-FAM) at the 5′ position. Double-stranded DNA was treated as previously described. The indicated amount of proteins was added to the well containing 12 nM of 6-FAM-labeled DNA in 20 mM sodium phosphate and 30 mM NaCl at pH 6.0. Reactions were measured six times by use of a SpectraMax Paradigm plate reader (Molecular Devices, CA, USA) with excitation wavelength 485 nm and emission wavelength 535 nm. Data were analyzed and plotted by use of GraphPad Prism 5 (San Diego, CA, USA).

### Isothermal titration calorimetry experiments

The samples of PmrA_C_ and box2-related DNAs were dialyzed overnight against the same reservoir of isothermal titration calorimetry (ITC) buffer [20 mM sodium phosphate, 30 mM NaCl and 0.5 mM Ethylenediaminetetraacetic acid (EDTA) at pH 6.0]. Box2b (1000 µM) was titrated into PmrA_C_ (75 µM) and 420 µM box2 was titrated into 100 µM PmrA_C_. Titrations of DNAs into buffer were used as control experiments. All titrations were performed on a MicroCal iTC200 microcalorimeter at 25°C. For each titration, 2 µl titrant was injected 15–20 times at 3-min intervals. Data were analyzed by use of Origin ITC Analysis (MicroCal Software, Northampton, MA, USA).

### Circular dichroism analysis

The purified PmrA_C_ protein (15 µM in 20 mM phosphate buffer) was analyzed at 25°C in a 1-mm path-length cuvette on an Aviv 202 CD spectrometer (Lakewood, NJ, USA) calibrated with d-10-camphorsulfonic acid. The steady-state circular dichroism (CD) spectra were recorded three times from 190 to 260 nm with wavelength steps of 0.5 nm and average time of 2 s or from 320 to 240 nm with wavelength steps of 0.5 nm and average time of 10 s. The equilibrium GdnHCl-denaturation experiment involved measuring the changes in CD signals at 216 nm from 0 to 6 M at 0.1 -M intervals and 2 min for equilibrium at 25°C. The denaturation curve was fitted to the two-state equation (28) as: *F* = {(α_N_ + β_N_[GdnHCl]) + (α_D_ + β_D_[GdnHCl]) exp[m([GdnHCl]− [D]^50%^)/RT]} / {1+ exp[m([GdnHCl]−[D]^50%^)/RT]}, where *F* is the CD signal; α_N_ is the CD signal at 0 M GdnHCl; β_N_ = dα_N_/d[GdnHCl]; α_D_ and β_D_ are the corresponding quantities for the denaturation state; [D]^50%^ is the GdnHCl concentration at which the protein is 50% unfolded; and m is the slope. The free energy of unfolding is given by ΔG = m × [D]^50%^. The CD spectra were displayed and analyzed by use of SigmaPlot 8.02 (SPSS Inc., Chicago, IL, USA).

### NMR and resonance assignment

All NMR spectra were acquired at 298 K on Bruker AVANCE 600, 800 or 850 MHz spectrometers equipped with a z-gradient TXI cryoprobe (Bruker, Karlsruhe, Germany). The NMR sample of PmrA_C_ consisted of 0.8 mM protein in 20 mM sodium phosphate and 30 mM NaCl at pH 6. For PmrA_C_–DNA complex samples, 0.2–0.5 mM PmrA_C_ and 2-fold of DNA were incubated in the same buffer of free PmrA_C_. The heteronuclear NMR spectra for resonance assignment of PmrA_C_ were obtained as described (29). Assignments of the main-chain ^15^N, ^1^H^N^, ^13^C^α^, ^13^C^β^ and ^13^C′ chemical shifts of PmrA_C_ and PmrA_C_–DNA complexes were based on NHCACB, CBCA(CO)NH, HNCO and HN(CA)CO spectra. Assignment of PmrA_C_ side-chain resonances was based on ^1^H-^15^N TOCSY-HSQC, ^1^H-^13^C HCCH-TOCSY, CC(CO)NH and HBHA(CO)NH spectra. Aromatic resonances of PmrA_C_ were assigned with use of 2D ^1^H-^13^C HSQC, HBCBCGCDHD, HBCBCGCDCEHE and NOESY spectra. The weighted chemical shift perturbations for backbone ^15^N and ^1^H_N_ resonances were calculated by the equation Δδ = {[(Δδ_HN_)^2^+(Δδ_N_/5)^2^]/2}^0.5^. To measure residual dipolar couplings, the filamentous bacteriophage Pf1 (8 mg/ml, Asla Biotech. Ltd., Latvia) was added into PmrA_C_ as the orienting medium and 2D ^1^H-coupled (F_1_) IPAP ^1^H-^15^N HSQC spectra were acquired with 256 complex *t_1_* (^15^N) points and 128 scans per *t_1_* increment for both the isotropic and anisotropic conditions. All NMR spectra were processed by use of NMRPipe (30) and analyzed by use of NMRView (31).

### PmrA_C_ structure calculation and analysis

Nuclear Overhauser effect (NOE) distance restraints were derived from a 3D ^15^N-edited NOESY-HSQC spectrum (150 ms mixing time) and ^13^C-edited NOESY-HSQC spectrum (150 ms mixing time). Peak intensities were classified as large, medium, small and very small, corresponding to upper-bound interproton distance restraints of 2.5, 3.5, 4.5 and 6.0 Å, respectively. An additional correction of 1.0 Å was added for methylene and methyl groups. Calculation of backbone φ, ψ torsion angles involved use of TALOS+ (32) and angles in good agreement with the NOE correlations were used for structure calculation. The solution structures for PmrA_C_ were determined with 1071 distance restraints, 82 hydrogen bonds restraints, 130 dihedral angle restraints, 67 ^1^D_NH_ residual dipolar coupling (RDC) constraints and the program XPLOR-NIH (33). The force constants and molecular parameters were set to default values as in the original sa_new.inp protocol in XPLOR-NIH. The backbone dihedral angles of the final converged structures were evaluated by the Ramachandran dihedral pattern of the PROCHECK-NMR program (34).

### HADDOCK docking

The information drive docking program HADDOCK 2.0 (35) was used to generate the PmrA_C_–box1 complex model. The starting structure for docking was a B-form model of the box1 DNA constructed with the InsightII package (Accelrys Inc., CA, USA) and the lowest energy structure of PmrA_C_. The residues with chemical shift perturbations of amide resonances >0.33 parts per million (ppm) and with high solvent accessibility (>50%) were selected as active residues and the neighbors of these active residues were selected as passive residues. For box1 DNA, THY4 to ADE11 and CYT16 to CYT22, which were all highly affected by titrating proteins (Figure 2C), were selected as active bases. However, the resulting models did not converge well due to the large range of bases for protein binding. To improve the docking, we need to lower the number of active bases. Based on the experimentally verified PmrA boxes in *Salmonella typhimurium* (In Supplementary Figure S1, *K. pneumoniae* PmrA shares 91% sequence identity to *Salmonella* PmrA for the C-terminal region Leu^151^ to Leu^216^), only CYT5 to THY10 and CYT16 to GUA21 were selected as active bases. Four kinds of relative orientations of PmrA_C_ molecules in complex with box1 DNA, head-to-head, head-to-tail, tail-to-head and tail-to-tail, were observed in the models. The best clusters from four orientations had similar HADDOCK scores and energies and we could not tell which orientation is preferred. To determine the protein orientation, we carried out NMR paramagnetic relaxation enhancement (PRE) study, which suggests the head-to-tail orientation. The HADDOCK models with head-to-tail orientation were carefully checked to find the possible bases that can interact with the three Arg side-chains and with Gly^211^ (which perturbed significantly in the presence of box1a). Finally, three sets of ambiguous interaction restraints (AIR) were defined. In the first set, the selected active residues were Lys^153^, Thr^187^, Asn^188^, Thr^189^, Glu^191^, His^193^ and Ile^194^ and the neighbors of these residues were selected as passive residues. CYT5 to THY10 and CYT16 to GUA21 were selected as active bases. In the second set, the AIR restraints were defined between the N_ε_H of Arg^171^, Arg^198^ and Arg^210^ and box1 from ADE8 to THY13 or ADE19 to ADE25. In the third set, the AIR restraints were defined as between the amide of Gly^211^ to the phosphate backbone of THY40 to ADE41 for half1 and THY28 to CYT30 for half2. Additional restraints to maintain base planarity and Watson–Crick base pairings were introduced for the DNA. During the rigid body energy minimization, 10 000 structures were calculated and the 200 best solutions based on the inter-molecular energy were selected for the semiflexible simulated annealing followed by explicit water refinement. The best 200 docked models were clustered by a cutoff of 3.5 Å, with a minimum of 10 structures in each cluster, which yielded six clusters. In terms of HADDOCK score and total energy, the best 10 structures from the first cluster were selected as the final models of the PmrA_C_–box1 complex.

### NMR paramagnetic relaxation enhancement experiments

Spin labeling of DNA was achieved by introducing the EDTA-conjugated deoxythymidine (dT-EDTA) into a DNA sequence as described (36). The box1 DNA sequences with dT-EDTA at THY4 or THY28 were from Midland Certified Inc. (TX, USA). Purity and authority of individual DNA strands were verified by chromatography and mass analysis. Double-stranded DNA was annealed as mentioned previously. For the paramagnetic state, DNA was mixed with an equal amount of MnCl_2_ and underwent dialysis overnight to remove the free Mn^2+^. For the diamagnetic state, CaCl_2_ was added into the DNA. For the complex sample with dT-EDTA at T4, the ratio of PmrA_C_ to box1 DNA was 1:1. For the sample with dT-EDTA at T28, twice the amount of PmrA_C_ was mixed with the box1 DNA. The ^1^H, ^15^N TROSY-HSQC spectra were acquired and the peak intensity of paramagnetic to diamagnetic state was calculated.

## RESULTS

### Confirmation of PmrA box on the *pbgP* promoter of *K. pneumoniae*

**Figure 1.**
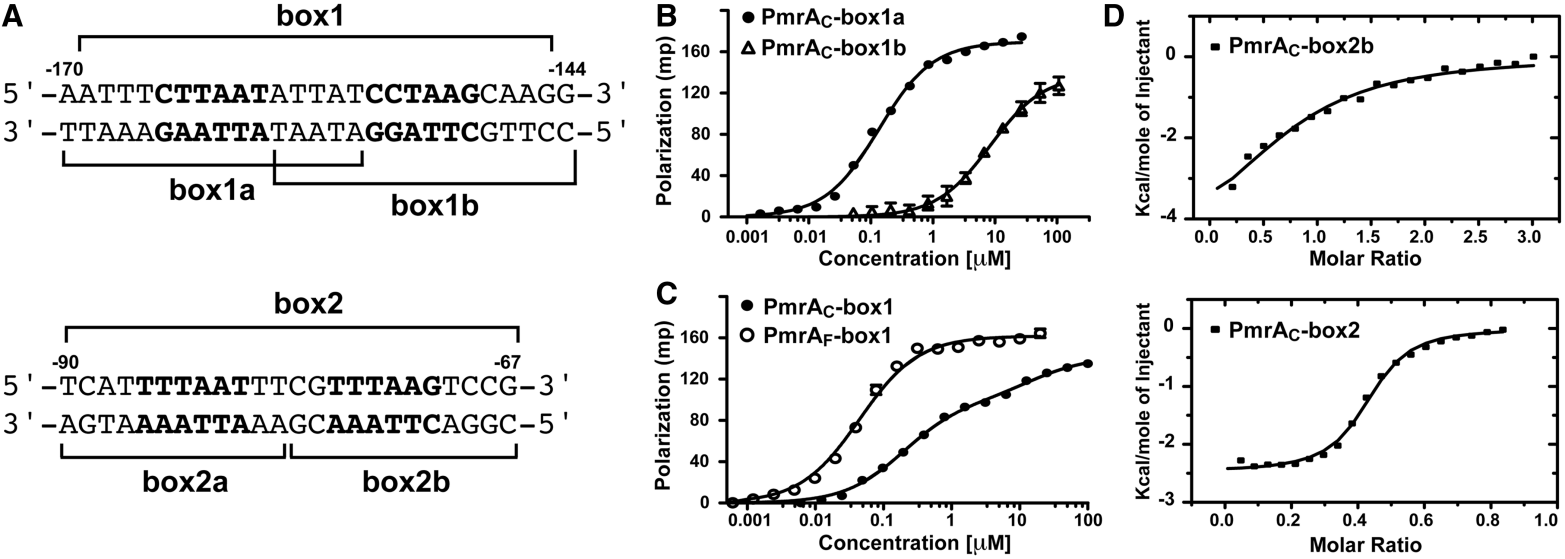
DNA recognition by PmrA_C_ or PmrA_F_. (**A**) The DNA sequences of box1 and box2 on the promoter region of *K. pneumoniae pbgP* gene. Their positions relative to the *pbgP* start codon are labeled. The first and second hexanucleotides are shown in bold and the synthesized DNA fragments are indicated. (**B**) Fluorescence polarization experiments of binding of PmrA_C_ and box1a or box1b sequence and (**C**) binding of PmrA_C_ or PmrA_F_ and box1 sequence. (**D**) and (**E**) Isothermal titration calorimetry of binding of PmrA_C_ and box2b and box2, respectively.

In *S. typhimurium*, the experimentally verified PmrA boxes, the DNA sequences PmrA recognizes, consist of a half1 site, ‘CTTAAG’ and a half2 site, ‘XTTAAT’ (X can be any nucleotide), separated by five base pairs (37). Also, DNase footprinting experiments with the PmrA protein from *Salmonella enterica* (In Supplementary Figure S1, *K. pneumoniae* PmrA shares 91% sequence identity to *Salmonella* PmrA for the C-terminal region Leu^151^ to Leu^216^) demonstrated specific binding to the *pbgP* promoter at the predicted PmrA box (TCTTAATATTATCCTAAGC, half1 and half2 sites underlined) (38). From these studies, we analyzed the sequence of the *K. pneumoniae* genome NTHU-K2044 (39,40) and identified a same PmrA box on the promoter of *pbgP* gene (at −170 position relative to the *pbgP* start codon), termed box1 (Figure 1A). At −90 position, we also identified another fragment, termed box2, which contains two possible binding sites, ‘TTTAAT’ and ‘TTTAAG’, separated by four base pairs (Figure 1A). To determine which fragment was the PmrA box, we synthesized six oligonucleotides, including box1, box2 and the sequences covering the half1 and half2 sites of box1 and 2, and characterized their binding affinity to PmrA_C_.

We used ITC experiments to investigate the interactions between PmrA_C_ and the six oligonucleotides. However, the measurement was hindered by severe aggregates during the process of titrating high-concentrated box1-related DNAs into PmrA_C_. We performed fluorescence polarization experiments to monitor the binding between fluorescence-labeled box1-related DNA sequences and PmrA_C_. The binding curves of PmrA_C_ with box1a and box1b were fitted by a single-site binding model. PmrA_C_ bound strongly to box1a with *K*_d_ 0.13 ± 0.01 µM and weakly to box1b with *K*_d_ 9.3 ± 1.5 µM (Figure 1B). The binding between PmrA_C_ and box1 (Figure 1C) showed a different binding curve, which qualitatively appeared as a two-site binding event. An extra-sum-of-squares *F*-test was performed to compare the goodness-of-fit of two-site and one-site binding models. The test showed that box1 contained two distinct PmrA_C_ binding sites (*F* = 146.7, *P* < 0.0001), with *K*_d_ 0.19 ± 0.01 and 15.1 ± 3.7 µM, similar to those with box1a and box1b binding, respectively, suggesting that PmrA_C_ binds to the two sites separately without cooperativity.

The thermodynamics of the interactions between PmrA_C_ and box2-related DNAs were successfully revealed by ITC, with the exception of box2a, which is strongly endothermic when titrating into buffer (data not shown). PmrA_C_ bound with 1:1 stoichiometry to box2b (Figure 1D) and with 2:1 stoichiometry to box2 (Figure 1E). The formations of the two complexes were both enthalpically driven and were fitted to the one-site binding model. The K_d_ values for PmrA_C_–box2b were 36.5 ± 9.4 µM and PmrA_C_–box2 0.97 ± 0.13 µM. The binding affinity was 37 times stronger to box2 than to box2b, which suggests positive cooperativity between the two PmrA_C_ molecules in box2 binding.

The interactions between PmrA_C_ and box2-related DNA were all weaker than those between PmrA_C_ and box1-related sequences, which agreed with foot-printing results (38). The box1 sequence, which contains two canonical PmrA_C_ binding sites separated by five base pairs, was verified to be the PmrA box on the promoter of *pbgP* gene from *K. pneumoniae*. However, the positive cooperativity in box2 binding implies that two PmrA_C_ molecules can have inter-molecular interactions to enhance DNA binding.

We investigated the interaction between PmrA_F_ and box1 by fluorescence polarization (Figure 1C) and found a one-site binding curve with *K*_d_ value 45.0 ± 2.3 nM, which is about 3-fold stronger than PmrA_C_ and box1a binding. Therefore, PmrA_F_ recognizes the two half-sites simultaneously and PmrA activation can increase the affinity for target DNA, which was suggested for several regulators from the OmpR/PhoB family (25,41,42) and confirmed here.

### PmrA_C_ and PmrA_F_ recognize tandem DNA with different modes

**Figure 2.**
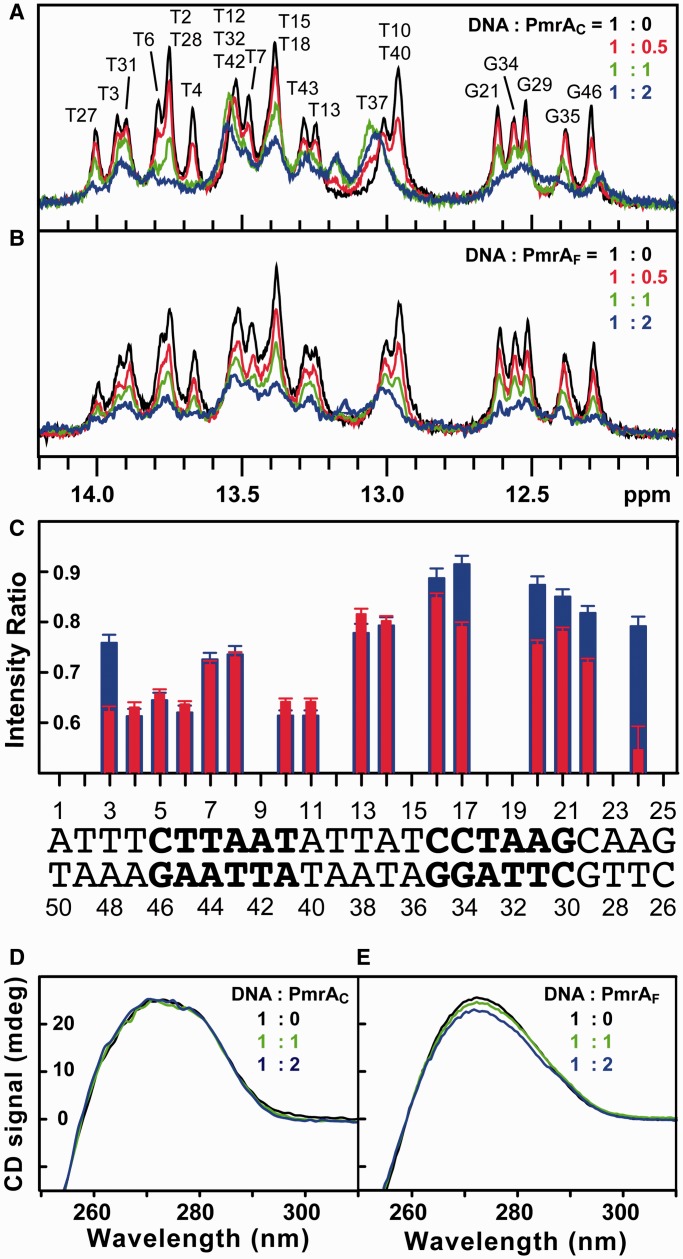
PmrA_C_ and PmrA_F_ recognize box1 DNA with different modes. (**A**) 1D proton NMR spectra of imino signals of box1 at different ratios of DNA to PmrA_C_ (black 1:0, red 1:0.5, green 1:1 and blue 1:2). The imino signals from THY3 to ADE24 were completely assigned. (**B**) 1D spectra of the titration of PmrA_F_ into box1 DNA. The color representation is the same as in (A). (**C**) The intensity ratio of each imino signal at ratio of protein to DNA of 0.5 to 0. Blue bars are for the PmrA_C_ complex and red are for the PmrA_F_ complex. The overlapped imino signals were not plotted and the sequence for box1 DNA is shown below. (**D**) and (**E**) CD spectra for box1 DNA at different ratios of DNA to PmrA_C_ and PmrA_F_, respectively.

To investigate the DNA recognition mode of PmrA_C_ and PmrA_F_, we examined the changes in DNA on protein binding by NMR 1D spectra. Figure 2A shows the 1D NMR spectra for imino protons of box1 at different ratios of PmrA_C_ to DNA. With ratio of protein to DNA (P/D) 0.5, the intensity of imino protons of DNA at the half2 site (e.g. GUA21, GUA29, GUA34 and GUA35) were similar to those for free DNA, but the intensity of those at the half1 site (e.g. THY6, THY7 and GUA46) was reduced significantly, which indicates that PmrA_C_ binds to half1 first. As the P/D ratio increased to 1, the imino signals for THY6 and GUA46 decreased to a minimum but did not decrease substantially when the ratio was increased to 2. However, the imino signals for half2 (GUA35, GUA34 and GUA21) continued to shift with P/D ratio from 1 to 2, which suggests that with this ratio, PmrA_C_ binds to the half2 site and this binding is in a fast exchange regime.

Differently, with titrations of PmrA_F_ into box1 (Figure 2B), the signals of all imino protons decreased with increasing P/D ratio, from 0 to 2, and the resonances of imino protons in the half2 site did not shift as severely as they did with PmrA_C_ titration. So unlike PmrA_C_, PmrA_F_ can form a stable contact with the half2 site. To clarify the differences between the two titrations, we plotted the intensity ratio for each imino signal at P/D ratio 0.5 to 0 (Figure 2C). The reduction in half1 imino signals was similar with both titrations, but the reduction in signals for half2 bases was greater with PmrA_F_ than PmrA_C_ titration. Also, the imino signals for THY3 and THY27 decreased severely only with the PmrA_F_ titration, which suggests that the binding of PmrA_F_ may bend or deform box1 DNA, thus leading to instability of base pairing in the 5′- and 3′-ends and the reduced THY3 and THY27 imino signals. NMR titrations showed that PmrA_C_ binds to only half1 site specifically and PmrA_F_ recognizes the two half-sites specifically and simultaneously, which agrees with the binding behavior probed by fluorescence polarization.

### Structural changes to DNA on protein binding

CD is a well-established technique for analysis of structural changes to DNA induced by protein binding (43). The CD spectra showed that PmrA_C_ binding caused almost no intensity change or wavelength shift in signals for box1 (Figure 2D). However, the CD signal at 273 nm decreases from 25.6 milli-degree (free box1) to 22.6 milli-degree (the errors are around 0.1 milli-degree) when 2-fold of PmrA_F_ is titrated into box1 (Figure 2E). Therefore, PmrA_F_ but not PmrA_C_ binding caused box1 DNA to bend or deform slightly, which agrees with the observations in NMR titration.

### Solution structure of PmrA_C_

**Figure 3.**
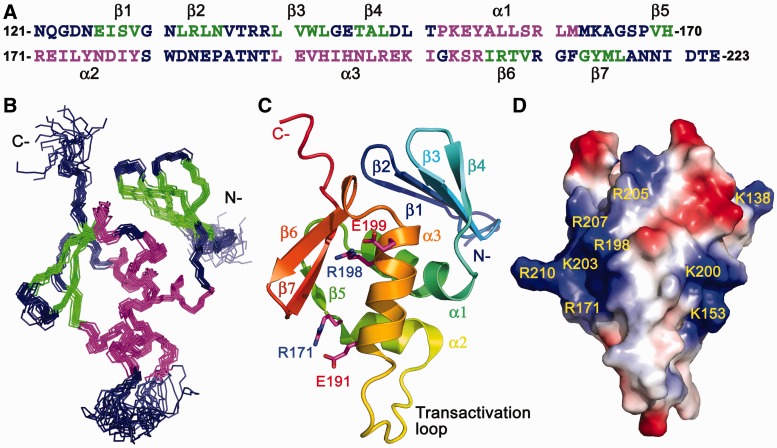
NMR solution structure of *K. pneumoniae* PmrA_C._ (**A**) Amino acid sequence of PmrA_C_ colored according to the secondary structures. The residues in β-strand are in green, α-helix in magenta and others in deep blue. (**B**) Backbone presentation of the 15 lowest energy structures with superimposition of the backbone atoms (H_N_, N, C_α_ and C’) in secondary structure regions. The residues are colored as in (A). (**C**) Secondary structures of the lowest energy structure of PmrA_C_ in rainbow color from the N-terminus (blue) to the C-terminus (red). Two salt bridges in α3 helix are shown as magenta sticks with nitrogen and oxygen atoms in blue and red, respectively. (**D**) Surface charge representations of PmrA_C_. Positively charged surface is in blue and labeled and negatively charged surface is in red.

CD spectra for PmrA_C_ at different pH values revealed that PmrA_C_ is well structured from pH 4.5 to 9.0 (Supplementary Figure S2A) The free energy of protein unfolding at pH 6.0 determined by GdnHCl denaturation followed by CD at 216 nm was 6.2 kcal/mol (Supplementary Figure S2B), which suggests that PmrA_C_ forms a stable conformation under this condition. Therefore, we acquired all NMR spectra with PmrA_C_ at pH 6.0. In total, 98% of backbone (only Asn^121^ is missing and not assigned) and 81% of side-chain atoms were assigned. The NMR structure of PmrA_C_ was calculated on the basis of 1071 distance restraints, 130 dihedral angle restraints and 67 RDC constraints by the simulated annealing protocol with the program XPLOR-NIH. In the final stage of refinement, we chose 15 structures with no NOE restraint violation >0.3 Å and no dihedral angle restraint >3° on the basis of lower total energy. The final 15 structures with a root mean square deviation (RMSD) 0.44 ± 0.08 Å for the backbone atoms and 1.10 ± 0.09 Å for the heavy atoms in the secondary structure regions are shown in Figure 3B. The structural statistics for these 15 structures are in Table 1. The fold of PmrA_C_ shows a winged helix motif consisting of a four-stranded antiparallel β-sheet (β1: Glu^126^-Val^129^, β2: Leu^132^-Asn^135^, β3: Leu^140^-Leu^143^ and β4: Thr^146^-Leu^148^), three α-helices (α1: Pro^152^-Met^163^, α2: Arg^171^-Tyr^179^ and α3: Leu^190^-Ile^201^), a short 3_10_ helix (Lys^203^-Arg^205^) and a C-terminal β-hairpin (β6: Ile^206^-Val^209^, and β7: Gly^213^-Leu^216^) flanked by a β-strand (β5: Val^169^-His^170^) (Figure 3A and B). In this structure, two negatively charged residues (Glu^191^ and Glu^199^) and one positively charged residue (Arg^198^) are located on the α3 helix, the DNA recognition helix. These charged residues are stabilized by the formation of salt bridges (Glu^191^-Arg^171^ and Glu^199^-Arg^198^) in the free state (Figure 3C). Surface charge presentation shows that the α3 helix is surrounded with several positively charged residues (Figure 3D).

**Table 1.** Structural statistics for the final 15 PmrA_C_ structures

*A. Constraints used*		
NMR restraints		
Intraresidue (|i-j| = 0)		299
sequential (|i-j| = 1)		350
medium range (|i-j| ≦ 4)		247
long range (|i-j| > 4)		175
total NOE distance restraints		1071
hydrogen bonds		41 × 2
dihedral angles		130
^1^D_HN_ RDC restraints		67
*B. Statistics for the Final X-PLOR Structures*		
No. of structures in the final set		15
X-PLOR energy (kcal.mol^−1^)		
E_NOE_		21.62 ± 2.54
E_cdih_		1.95 ± 0.36
E_bond_ + E_angle_ + E_improper_		104.25 ± 6.42
E_VDW_		68.28 ± 5.31
Mean global root mean square deviation (Å)		
Backbone (*N*, C^α^, C’)		
Residues	α-helix: 152–163, 171–179, 190–201	0.44 ± 0.08
	β-strands: 126–129, 132–135, 140–143, 146–148, 169–170, 206–209, 213-216	
Heavy atoms		
Residues	α-helix: 152–164, 171–178, 190–201	1.10 ± 0.09
	β-strands: 126–129, 132–135, 140–143, 146–148, 166–169, 206–209, 213-216	
Ramachandran data		
Residues in most favored regions (%)		76.9
Residues in allowed regions (%)		18.5
Residues in generously allowed regions (%)		4.5
Residues in disallowed regions (%)		0.1

### Mapping the DNA interaction site of PmrA_C_

**Figure 4.**
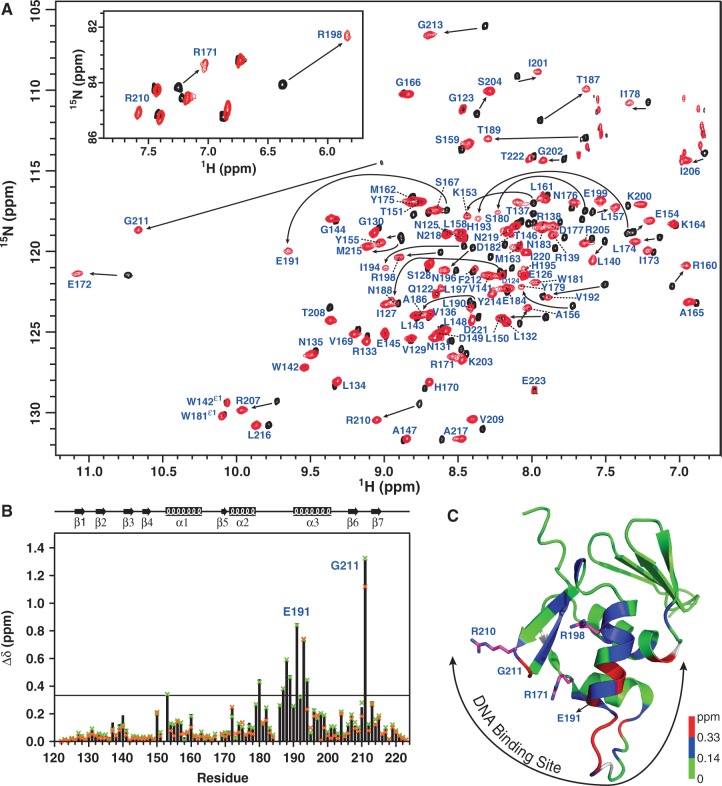
NMR investigations of PmrA_C_–DNA complexes. (**A**) The regions of amide resonances and N_ε_H resonances of Arg side-chains (inset) of overlaid 2D ^1^H, ^15^N TROSY-HSQC spectra for PmrA_C_ in the absence (black) or presence (red) of box1a DNA. The amide resonances in complex state are indicated. (**B**) Weighted chemical shift perturbations for backbone ^15^N and ^1^H_N_ resonances as calculated by the equation Δδ = {[(Δδ_HN_)^2^+(Δδ_N_/5)^2^]/2}^0.5^. The solid black bar represents the Δδ values for the box1a complex, green x for box1 and orange x for box1b. The black line indicates 0.33 ppm (the mean Δδ value of box1a complex plus 1 SD). (**C**) Structural mapping of chemical shift perturbations of the box1a complex. The residues with chemical shift perturbation >0.33 ppm are in red, <0.14 ppm (the mean Δδ value of the box1a complex) green and 0.14 to 0.33 ppm blue. The proline residues and the residues without data are in white. The top two most-perturbed residues, Gly^211^ and Glu^191^, are indicated. Side-chains of the three Arg residues are shown as magenta sticks with nitrogen atoms in blue. The DNA-binding site of PmrA_C_ consists of the α3 helix, the transactivation loop, the C-terminal β-hairpin and adjacent residues.

We detected the DNA-binding site of PmrA_C_ by measuring the chemical shift perturbations of backbone amide resonances and N_ε_H resonances of the Arg residues (Figure 4A) of PmrA_C_ with box1a binding. The weighted chemical shift perturbations of backbone amide resonances (Figure 4B) were calculated and mapped onto the PmrA_C_ structure (Figure 4C). The backbone amide resonances with significant chemical shift perturbations on box1a binding (Δδ > Δδ_average_ + SD ∼ 0.33 ppm) were mostly located at the α3 helix and the transactivation loop between α2 and α3. Also, the amide of the wing residue, Gly^211^, showed the most significant downfield shift. In the PhoB_C_–DNA crystal structure, the amide proton of the corresponding Gly residue forms an H-bond with the DNA phosphate backbone. Accordingly, the significant downfield shift of amide resonances of Gly^211^ for PmrA_C_ in the complex state may originate from the de-shielding effect of the H-bond formation.

In addition to backbone amides, the N_ε_H resonances of three Arg side-chains were extensively perturbed in the presence of box1a DNA (inset in Figure 4A). The N_ε_H resonances of Arg^171^ and Arg^198^, which were stabilized by salt bridges in the free state, shifted upfield in the presence of box1a DNA, which implies that the salt bridges were disturbed by the DNA. The N_ε_H resonances of Arg^210^, missing in the free form, were detected in the complex state, which suggests that the interaction between N_ε_H and DNA decreases the exchange rate with water. From the perturbations of backbone amide resonances and Arg N_ε_H resonances, the DNA-binding site of PmrA_C_ consists of the α3 helix, the transactivation loop, the C-terminal β-hairpin and some residues adjacent to these regions (Figure 4C).

We detected the interactions between PmrA_C_ and box1b or box1 DNA sequences by NMR. For the PmrA_C_–box1b complex, several residues in the α3 helix disappeared and the weighted chemical shift perturbations of most of the residues were smaller than those for the PmrA_C_–box1a complex (Figure 4B). Interestingly, the PmrA_C_–box1 complex, with ratio of protein to box1 of 2:1, contained only one set of protein amide resonances, which were very close to those in the PmrA_C_–box1a complex (Figure 4B). We did not detect resonances from PmrA_C_ binding with the half2 site, which may due to the intermediate exchange between PmrA_C_ and the half2 site DNA.

We also investigated the binding between PmrA_C_ and box2-related DNA sequences by NMR titration. Upon titration of box2b DNA into ^15^N-labeled PmrA_C_, backbone amide resonances moved continuously (Supplementary Figure S3A), which suggests that the binding was on a fast-exchange time scale. We used the titration curves for PmrA_C_ residues with significant chemical shift perturbations to determine the *K*_d_ values of box2b binding (Supplementary Figure S3B), which were in the range of 28.7–35.5 µM, resembling the values from ITC analysis. For the PmrA_C_–box2 complex, the residues in the transactivation loop and the α3 helix were missing, suggesting intermediate exchange. We plotted the weighted chemical shift perturbations for backbone amide resonances between the free and complex states with box2b and box2 in the function of residue number (Supplementary Figure S3C). In PmrA_C_–box2 complex, the residues at the α2 helix, α3 helix and C-terminal β-hairpin changed significantly but this pattern of shift is quite different with the shift of box1 binding, suggesting that PmrA_C_ binds to box1 and box2 with different orientations. Also, the changes in chemical shift were much smaller than for the box1 complex (Figure 3A) and the amide resonance of Gly^211^ could not be identified in the complex with box2b and box2 DNA, which suggests that Gly^211^ could not form a stable H-bond with the phosphate backbone of box2 DNA. PmrA_C_ may bind non-specifically to box2 DNA, which agrees with *K*_d_ findings.

### The model of the PmrA_C_–box1 complex

To gain insights into the structural basis of DNA recognition by PmrA_C_, we generated a model of the PmrA_C_–box1 complex using HADDOCK (35). The process of HADDOCK docking is described in ‘Materials and Methods’ section. Briefly, the lowest energy NMR structure of PmrA_C_ and the B-form DNA model of the box1 sequence were used as initial structures for modeling the protein–DNA complex. We defined two half-site hexanucleotides as active bases. The active residues of PmrA_C_ were defined as those with weighted chemical shift perturbation >0.33 ppm (Δδ_average_ + SD ∼ 0.33 ppm) and high solvent accessibility (>50%). In addition, we defined AIR restraints between the amide proton of Gly^211^ and the phosphate backbone of DNA and between the side-chains of Arg^171^, Arg^198^ and Arg^210^ and the phosphate backbone or base of DNA.

**Figure 5.**
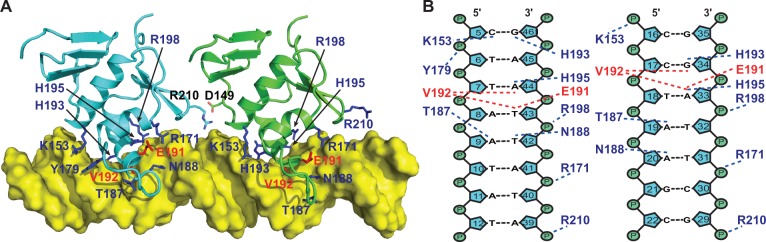
Structural model of PmrA_C_–box1 DNA complex. (**A**) The best complex model from HADDOCK docking with yellow surface display of DNA and ribbon presentation of PmrA_C_, showing that the α3 helix and the C-terminal β-hairpin fit into the major and minor groove of the DNA, respectively. The residues showing H-bond and non-bonded interactions with DNA are in blue and red, respectively. The inter-molecular salt bridge identified between the two PmrA_C_ molecules is shown. (**B**) Schematic presentation of the detailed interactions between the two PmrA_C_ molecules and box1 DNA identified in more than five of the best 10 models. The H-bonds and non-bonded contacts are indicated by blue and red dotted lines, respectively.

After the HADDOCK docking protocol, the final 200 water-refined complex structures were clustered based on the pair-wise RMSD matrix using a 3.5 Å cutoff and resulted in six different clusters. Supplementary Figure S4 shows the top 10 structures from each cluster and their structural statistics. Cluster 1 is the best in terms of HADDOCK score and energy. The 10 structures with the lowest energy from this cluster were selected to examine possible interactions between PmrA_C_ and box1. Basically, the recognition helix α3 is inserted into the major grooves of two half-site hexanucleotides for specific recognition and the wing contacts the DNA in the minor groove (Figure 5A). We plotted the inter-molecular H-bonds and hydrophobic interactions observed in more than 5 of the final 10 structures (Figure 5B). Basically, the side-chains of Lys^153^, Thr^187^, Asn^188^, His^193^ and His^195^ were responsible for DNA-specific H-bond interactions and the side-chains of Arg^171^, Arg^198^ and Arg^210^ formed H-bonds with the DNA phosphate backbone. Non-bonded contacts were found between Glu^191^, Val^192^ and box1. The amide proton of Gly^211^, although not forming an H-bond, is close to the backbone phosphate. Interestingly, we observed an inter-molecular salt bridge between Arg^210^ (PmrA_C_ at half1) and Asp^149^ (PmrA_C_ at half2) in 6 of the final 10 models, although we did not add any restraints. The observation of many inter-molecular interactions between PmrA_C_ and half1 DNA agrees well with the low *K*_d_ value (0.19 ± 0.01 µM) for this complex. Also, the model showed that the two PmrA_C_ molecules are too far away to form stable contacts when binding to straight box1 DNA.

**Figure 6.**
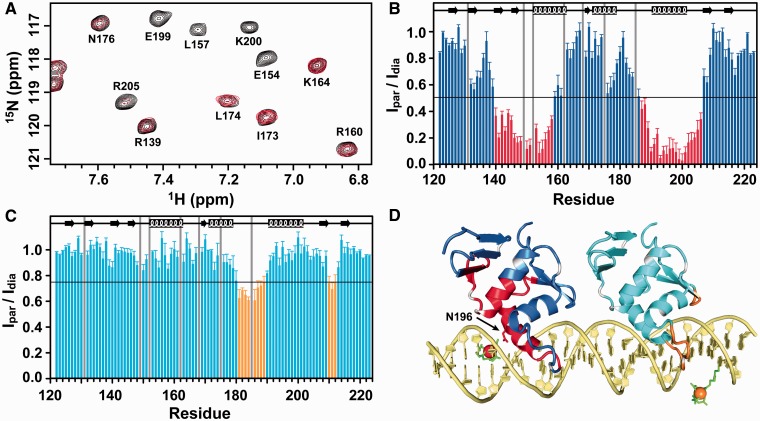
Inter-molecular PRE for the PmrA_C_–box1 DNA complex. (**A**) A portion of 2D ^1^H, ^15^N TROSY-HSQC acquiring in the paramagnetic state (in red; EDTA is chelated with Mn^2+^) overlaid with that in the diamagnetic state (in black; EDTA is chelated with Ca^2+^) for PmrA_C_–box1 complex with dT-EDTA at THY4. (**B**) and (**C**) The ratios of peak intensity from the paramagnetic to diamagnetic state with dT-EDTA at THY4 and THY28, respectively. The proline and the severely overlapped residues are shown with gray bars. In (B), the residues with intensity ratio <0.5 are in red. In (C), the residues with intensity ratio <0.75 are in orange. (D) The best structural model from HADDOCK colored according to the PRE results. For spin-labeling at THY4, the Mn^2+^ and residues with intensity ratio <0.5 are in red. For spin-labeling at THY28, the Mn^2+^ and the residues with intensity ratio <0.75 are in orange. The EDTA is shown with green sticks.

To validate the HADDOCK model, we recorded the NMR inter-molecular PRE effects for ^15^N, ^2^H-labeled PmrA_C_ in the presence of box1 DNA with spin-labeling. We purchased two box1 sequences with dT-EDTA at THY4 or THY28 (Midland Certified Inc., TX, USA). In preparing the complex sample for spin-labeling at THY4, the amount of PmrA_C_ to box1 was set at 1 to 1. With spin-labeling at THY28, box1 was incubated with twice the amount of PmrA_C_. The TROSY-HSQC spectra for two complex samples were acquired at the paramagnetic state (EDTA chelated with Mn^2+^) and diamagnetic state (EDTA chelated with Ca^2+^) (Figure 6A) and these spectra superimposed well with the spectra from complex sample without spin-labeling on DNA, suggesting spin-labeling at THY4 or THY28 does not affect the protein–DNA interaction. The proportion of peak intensities measured as paramagnetic state to diamagnetic state (I_par_/I_dia_) were calculated (Figure 6B and C) and mapped on the HADDOCK complex structure (Figure 6D). With spin-labeling at THY4, the residue closest to Mn^2+^ was Asn^196^ (∼20 Å), with an I_par_/I_dia_ value around zero. The amide intensities of residues near Asn^196^ were also severely attenuated. The PRE effects were smaller with spin-labeling at THY28 than at THY4, because with the former, PmrA_C_ bound to the half1 site was too far to be affected by the spin-labeling and the binding affinity of PmrA_C_ to the half2 site was weak. In the complex with spin-labeling at THY28, I_par_/I_dia_ values were significantly decreased for residues Trp^181^ to Asn^188^ and Arg^210^ to Phe^212^ and the distances between these residues to Mn^2+^ chelated by EDTA at THY28 were all <31 Å. In summary, the PRE effects from spin-labeling at two different bases agreed well with the complex structure generated by HADDOCK docking.

### Box1 DNA recognition by PmrA_F_

**Figure 7.**
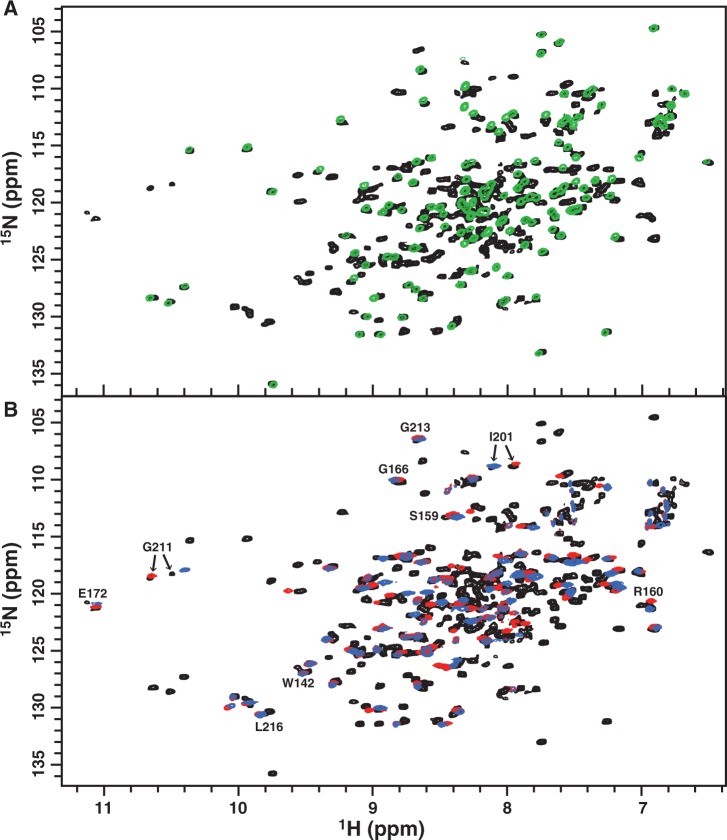
NMR study of the PmrA_F_–box1 DNA complex. (**A**) 2D ^1^H, ^15^N TROSY-HSQC spectra for the PmrA_F_–box1 complex with ratio of protein to DNA of 2:1 shown in black and that of BeF3^−^-activated PmrA_N_ in green. The good superimposition of the two spectra indicates that the N-terminal domain of PmrA_F_ in the complex state shows the free-state-like homodimeric conformation. (**B**) 2D ^1^H, ^15^N TROSY-HSQC spectra for the PmrA_F_–box1 complex (black) overlaid with that of PmrA_C_–box1a complex (red) and PmrA_C_–box1b complex (blue). Two sets of resonance peaks were observed for a number of residues in the C-terminal domain of PmrA_F_ in complex with box1 and some are labeled.

To understand how dimerization of the N-terminal receiver domain of the response regulator can enhance the recognition of the C-terminal effector domain to its target DNA, a structure of the full-length response regulator in complex with DNA is needed. The protein data bank contains many structures of C-terminal effector domains with and without DNA, yet no structure of full-length response regulator bound to DNA has been published. Hence, in addition to studying PmrA_C_, we also investigated the interaction between PmrA_F_ and box1 DNA by NMR. We successfully prepared the sample of ^2^H-, ^13^C- and ^15^N-labeled PmrA_F_ bound to box1 with P/D ratio of 2. The ^1^H, ^15^N TROSY-HSQC spectrum for this complex revealed one set of resonance peaks from the N-terminal receiver domain, which superimposed well with the spectrum for BeF_3_^−^-activated PmrA_N_ (Figure 7A), which indicates that the N-terminal domain of PmrA_F_ maintains an activated homodimeric conformation comparable with the structure of BeF_3_^−^-activated PmrA_N_ in the absence of PmrA_C_ and DNA (27). As well, the N-terminal and C-terminal domains do not seem to interact extensively, so DNA binding does not significantly perturb the N-terminal domain.

Unlike the PmrA_C_–box1 complex, which exhibits only one set of resonances similar to those in the PmrA_C_–box1a complex, the C-terminal effector domain of the PmrA_F_–box1 complex showed two sets of resonance peaks for plenty of residues. We overlaid this complex spectrum with those from PmrA_C_–box1a or PmrA_C_–box1b complexes and found that they were similar (Figure 7B). For example, one of the Gly^211^ resonances superimposed well with the peak from the PmrA_C_–box1a complex and another peak deviated slightly from that for the PmrA_C_–box1b complex, which suggests that one C-terminal effector domain binds to the half1 site and another domain recognizes the half2 site. Also, Leu^216^, which exhibits overlapped amide resonances in complex with box1a and box1b, showed two amide resonances in the presence of box1. Similar spectra were also observed on Trp^142^. The two residues are distant from the DNA-binding site and two sets of resonances may be caused by asymmetrical interactions between two tandem C-terminal domains in a head-to-tail arrangement when binding to box1. Therefore, in the PmrA_F_ dimer, the N-terminal domain keeps a free-form-like conformation and the inter-domain interactions between two tandem C-terminal domains may increase their binding affinity to the two half-sites on the box1 promoter DNA.

The backbone resonance assignment of PmrA_F_ in complex with box1 DNA is not completed yet because of the molecular weight of this complex (∼70 kDa) and the severe overlapping of peaks. However, the two sets of resonances observed on the C-terminal domain residues indicate that NMR can detect the difference between the half1- and half2-bound structures, which implies the feasibility of NMR structure determination, along with X-ray crystallography.

## DISCUSSION

### Structural features of PmrA_C_ and PmrA_C_–box1 complex

*Klebsiella pneumoniae* PmrA_C_ has the structural topology of β1 -β2-β3-β4-α1-β5-α2-α3-β6-β7, which is typically observed for proteins in the OmpR/PhoB superfamily. Search of the DALI database (44) produced hundreds of hits with *z*-scores > 2.0; the top three were for DrrD (*z*-score = 10.7) (19), YycF (*z*-score = 10.6) (45) and PhoP (*z*-score = 10.5) (46). The *z*-scores for the two well-studied effector domains, PhoB (23) and OmpR (47), were 9.5 and 7.7, respectively. The sequence alignment and structural comparison of PmrA_C_ with these proteins are in Supplementary Figure S5A and B, respectively. These proteins share only 8% sequence identity but their structures are similar. The superimposition of Cα atoms of secondary structural regions gives RMSD values of 1.76, 2.22, 2.06, 2.34, 2.39 and 2.83 Å between PmrA_C_ and DrrD (1KGS), YycF (2D1V), PhoP (2PMU), PhoB (1GXQ), PhoB–DNA complex (1GXP) and OmpR (1OPC), respectively. Interestingly, the small 3_10_ helix after α3 was observed only for PmrA_C_ and PhoB_C_ but not other effector domains.

In PhoB, the loop connecting α2 and α3, also called the transactivation loop, was found to be important for interacting with the RNA polymerase holoenzyme (48). Four mutants (W184R, G185R, V190M and D192G) in this loop were found to abolish the activation of transcription and three of them were involved with charged residues. Recently, the complex structure for σ_4 _-β-flap/PhoB_C_–pho box DNA was determined, revealing that an acidic patch (Glu^177^ and Glu^191^) on the transactivation loop of PhoB_C_ faces a patch of basic residues from the σ_4_ helix α4 (49). From these studies, the acidic patch at the transactivation loop was found to be important for transcription activation by PhoB. In the structure of *K. pneumoniae* PmrA_C_, the transactivation loop also formed an acidic patch by Glu^172^, Asp^182^ and Glu^184^ (Supplementary Figure S5A), so the mechanism of transcription activation by *K. pneumoniae* PmrA may be similar to that for PhoB.

The HADDOCK model of the PmrA_C_–box1 DNA complex reveals a good complementary fit between PmrA_C_ and the major groove of DNA and suggests several residues for base-specific interactions (Figure 5A). The α3 recognition helix has a major role in interacting with distinct DNA sequences among winged-helix effector domains. Therefore, the key residues for specific DNA binding may be derived by sequence alignment (Supplementary Figure S5A), in which, the PmrA residues His^193^, His^195^, Asn^196^ and Glu^199^ on α3 are not conserved, suggesting that they may contribute to base-specific recognition. In the crystal structure of the PhoB_C_–DNA complex (23), Arg^201^ forms a specific H-bond with guanine and Thr^194^ and Val^197^ form van der Waals contacts with thymines in both half-sites. Another specific H-bond is identified between Arg^219^ and bases between two half-sites. Other residues on PhoB_C_ form salt bridges or H-bonds with DNA phosphate backbone (Supplementary Figure S5C). In PmrA, similar van der Waals contacts are observed and Arg^210^ (the corresponding residue of Arg^219^) recognizes DNA phosphate backbone (Figure 5). However, we cannot identify any interaction between Asn^196^ (the corresponding residue of Arg^201^) and the DNA in two half-sites. Instead, in our model the residues His^193^ and His^195^ form H-bond interactions with the bases ADE44 and THY43, respectively. From the sequence alignment and the comparison with the PhoB_C_–DNA complex, the residues His^193^ and His^195^ are highly likely to be the determinants of PmrA base specificity.

The arrangement of effector domains bound with two half-sites is divergent. The PhoB effector domain binds to DNA as a head-to-tail dimer (23) and that of OmpR can contact DNA in both head-to-tail or head-to-head orientations (24,25). In the PhoB_C_–DNA complex structure (23), the DNA bends by protein binding, with extensive protein–protein contacts between the C-terminal β-hairpin and C-terminal tail of the upstream protein and the N-terminal β-sheet of the downstream protein (head-to-tail). For the PmrA_C_–box1 complex, with successful spin-labeling on box1 DNA, we concluded that two PmrA_C_ molecules bound to the two half-sites in a head-to-tail fashion (Figure 6). Moreover, in the HADDOCK complex model (Figure 5), we observed an inter-molecular salt bridge between Arg^210^ (in the C-terminal β-hairpin of PmrA_C_ at half1) and Asp^149^ (in the end of the N-terminal β-sheet of PmrA_C_ at half2). Therefore, although *K. pneumoniae* PmrA has different residues for DNA-specific binding, the head-to-tail domain arrangement for DNA recognition and the property of a transactivation loop are similar to those for PhoB, so the two proteins may have a similar mechanism of transcription activation.

### How activation of PmrA enhances DNA recognition

Despite the abundance of information regarding the function of OmpR/PhoB RRs, a detailed picture of how activated RRs bind to DNA and activate transcription is lacking. In the common activation mechanism of OmpR/PhoB RR, the phosphorylation of the conserved Asp residue triggers the formation of a head-to-head dimer of the N-terminal receiver domain, which will enhance the binding of the C-terminal effector/DNA-binding domain to the imperfect or perfect tandem repeat sequences on the promoters of target genes to activate transcription. In this study, we demonstrate enhanced DNA recognition with the full-length PmrA activated by the phosphoryl analog BeF3^-^. The binding between PmrA_C_ and box1 was first measured by fluorescence polarization, showing a two-site binding curve with two *K*_d_ values similar to those with box1a and box1b binding (Figure 1C); therefore, PmrA_C_ binds to the two half-sites separately without any cooperation. As well, the binding was weaker to the half2 than half1 site. The 1D NMR titration experiments further demonstrated that PmrA_C_ binds first to the half1 then the half2 site (Figure 2A). However, for the interaction between PmrA_F_ and box1, we found a one-site binding curve (Figure 1C), with 3-fold stronger binding affinity than between PmrA_C_ and box1a. The 1D NMR titration experiments also showed that PmrA_F_ recognizes the two half-sites specifically and simultaneously. Why PmrA_C_ binds weakly to box1b (*K*_d_ = 9.3 ± 1.5 µM) but the same C-terminal domains of PmrA_F_ bind to the two half-sites with much stronger affinity (*K*_d_ = 45.0 ± 2.3 nM) remains unclear. Do the two C-terminal domains cooperate in PmrA_F_–DNA binding?

In the binding of PmrA_C_ to box2, the binding affinity of PmrA_C_ was 37 times stronger to box2 than box2b (Figure 1D and E), which demonstrates positive cooperativity between the two PmrA_C_ molecules. The absence of cooperativity in box1 binding and the positive cooperativity in box2 binding suggests that the cooperativity arises from the shorter nucleotide spacing (5 base pairs in box1 DNA and 4 base pairs in box2 DNA), which allows for extensive inter-molecular interactions between the two PmrA_C_ molecules. For the PmrA_F_–box1 complex, although the two half-sites are separated by 5 base pairs, CD studies suggested that the DNA is slightly bent (Figure 2D), which shortened the distance between the two tandem PmrA_C_ molecules bound with box1. Our HADDOCK model revealed one inter-molecular salt bridge between two PmrA_C_ molecules bound with un-bending DNA. Also, the ^1^H, ^15^N TROSY-HSQC spectra for PmrA_F_ in complex with box1 showed that the DNA-recognition residues as well as those far away from DNA-binding site exhibit two resonance peaks (Figure 7B) that may originate from the asymmetrical interactions between two tandem C-terminal domains in a head-to-tail orientation when binding to box1. All these results suggest that the cooperativity is from the inter-domain interactions between two C-terminal domains.

In conclusion, we propose the structural events of PmrA activation and promoter DNA binding. PmrA_C_ prefers the 5′-CTTAAT-3′ sequence to 5′-CCTAAG-3′ and 5′-TTTAAG-3′ sequences. The phosphorylation of PmrA triggers the formation of a head-to-head dimer in the N-terminal domain by use of a conserved α4 -β5-α5 interface, and this conformation is not disturbed by DNA binding. However, the formation of a dimer brings the two C-terminal domains close to each other to recognize the two half-sites of box1 DNA simultaneously and specifically in a head-to-tail fashion. The residues Lys^153^, Thr^187^, Asn^188^, His^193^ and His^195^ are involved in DNA-specific recognition and Arg^171^, Arg^198^ and Arg^210^ are responsible for interactions with the DNA phosphate backbone. Also, the C-terminal β-hairpin and C-terminal tail of the upstream PmrA_C_ closely contact the N-terminal β-sheet of the downstream PmrA_C_. These interaction networks bend the DNA slightly, and a stable PmrA_F_–box1 DNA complex is formed to activate transcription.

## ACCESSION NUMBERS

The chemical shifts of PmrA_C_ at pH 6.0 and 298 K were deposited into BioMagResBank under accession number BMRB-19231. The ensemble of 15 NMR structures and the averaged structure, along with the complete list of restraints, were deposited in the RCSB Protein Data Bank under accession number 2m87.

## SUPPLEMENTARY DATA

Supplementary Data are available at NAR Online.

## FUNDING

Academia Sinica, the Ministry of Education under the ATU plan and the National Science Council Taiwan, ROC [NSC 100-2311-B-001-025-MY3]. Funding for open access charge: Academia Sinica and National Science Council [NSC 100-2311-B-001-025-MY3], Taiwan, ROC.

*Conflict of interest statement*. None declared.

## Supplementary Material

Supplementary Data
