# Supplementary material for: Solution structure and tandem DNA recognition of the C-terminal effector domain of PmrA from Klebsiella pneumoniae
Source: Nucleic Acids Res. 2013 Dec 25;42(6):4080–93. doi: 10.1093/nar/gkt1345 (PMC3973317; doi:10.1093/nar/gkt1345)
Supplement: Supplementary Data [file supp_42_6_4080__index.html]

Solution structure and tandem DNA recognition of the C-terminal effector domain of PmrA from Klebsiella pneumoniae — Supplementary Data 

# Solution structure and tandem DNA recognition of the C-terminal effector domain of PmrA from *Klebsiella pneumoniae*

## Supplementary Data

files

**Files in this Data Supplement:**

- Supplementary Data - pdf file
