# Supplementary material for: Solution structure and tandem DNA recognition of the C-terminal effector domain of PmrA from Klebsiella pneumoniae
Source: Nucleic Acids Res. 2013 Dec 25;42(6):4080–93. doi: 10.1093/nar/gkt1345 (PMC3973317; doi:10.1093/nar/gkt1345)
Supplement: Supplementary Data [file supp_gkt1345_nar-03007-f-2013-File009.pdf]

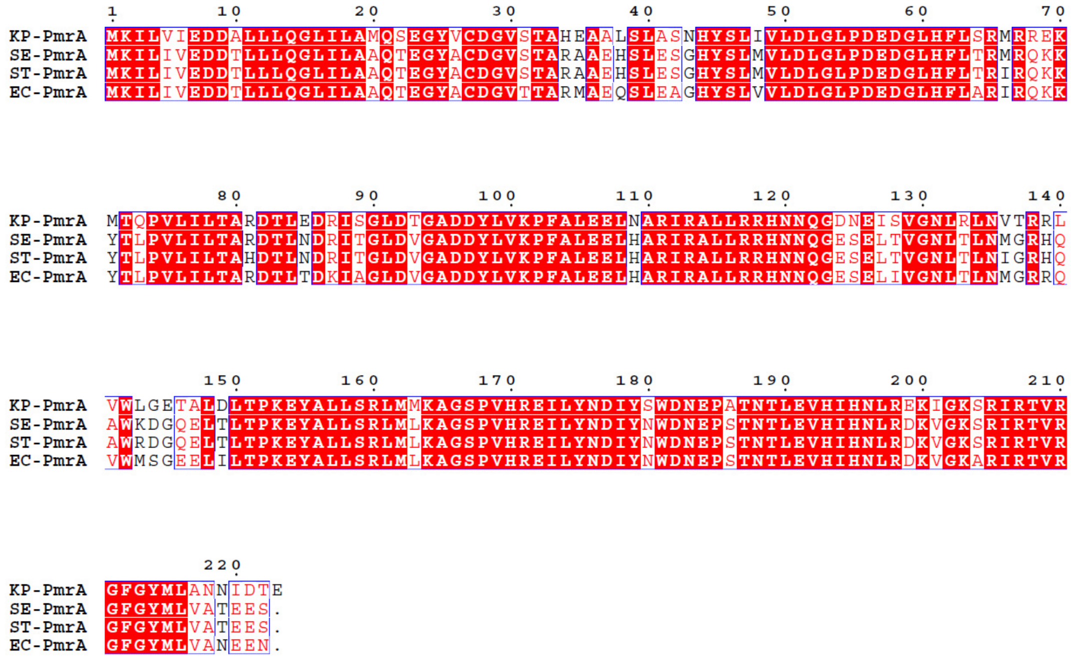

Supplementary Figure S1. Sequence alignment of PmrA proteins from *Klebsiella pneumoniae* (KP), *Salmonella enterica* (SE), *Salmonella typhimurium* (ST) and *Escherichia coli* (EC).

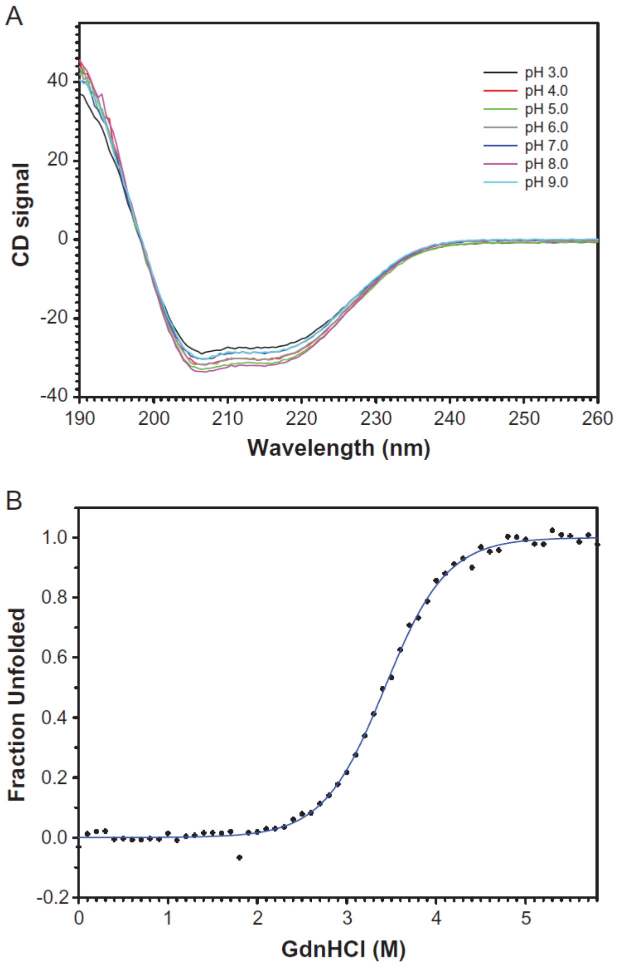

Supplementary Figure S2. Circular dichroism investigation on PmrA_C. (A) Far-UV CD spectra of *K. pneumoniae* PmrA_C at different pH values. (B) Gdn-HCl denaturation experiment of PmrA_C at pH 6.0 followed by CD at 216 nm. The denaturation curve was fitted to the two-state equation: $F = \{(\alpha_N + \beta_N[\text{GdnHCl}]) + (\alpha_D + \beta_D[\text{GdnHCl}]) \exp[m([\text{GdnHCl}]-[D]^{50\%})/RT]\} \ / \ \{1+ \exp[m([\text{GdnHCl}]-[D]^{50\%})/RT]\}$, where F is the CD signal; α_N is the CD signal at 0M GdnHCl; $\beta_N = d\alpha_N/d[\text{GdnHCl}]$; α_D and β_D are the corresponding quantities for the denaturation state; $[D]^{50\%}$ is the GdnHCl concentration at which the protein is 50% unfolded; and m is the slope. The free energy of unfolding is given by $\Delta G = m \times [D]^{50\%}$.

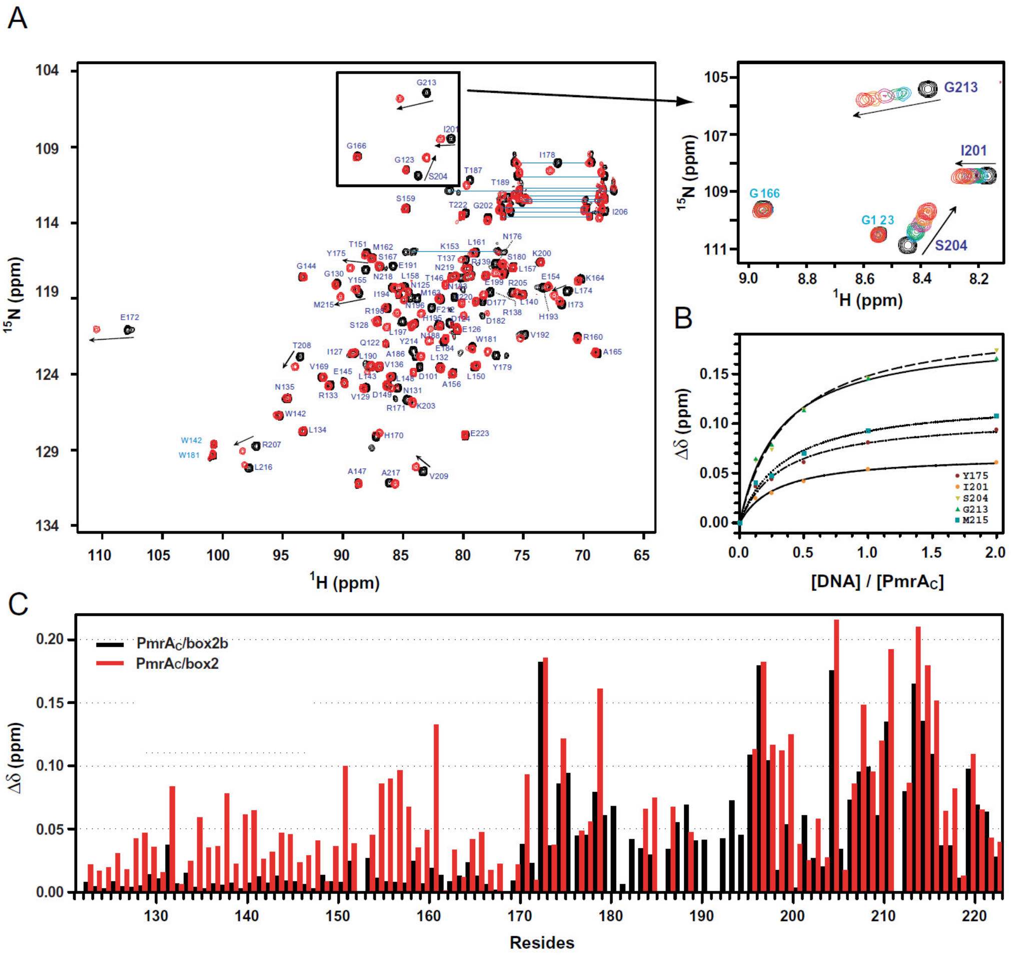

Supplementary Figure S3. NMR analysis of interactions between $PmrA_C$ and box2-related DNAs. (A) The superimposed ^{1}H-^{15}N TROSY-HSQC spectra of free (black) and box2b bound $PmrA_C$ (red). A selective section shows the spectra of $PmrA_C$ acquired on the titration of an increasing amount of box2b DNA (DNA to protein ratios is 0 (black), 0.125 (cyan), 0.25 (green), 0.5 (purple), 1.0 (orange), and 2.0 (red), respectively). (B) Titration profiles for several residues of $PmrA_C$ were plotted as a function of the DNA/$PmrA_C$ ratio. Their dissociation constants were calculated by a single-site binding model. (C) Weighted chemical shift perturbations for backbone ^{15}N and ^1H_N resonances as calculated by the equation $\Delta\delta = [((\Delta\delta_{HN})^2+(\Delta\delta_N/5)^2)/2]^{0.5}$. The black bar represents the $\Delta\delta$ values of the box2b complex and red for box2.

A

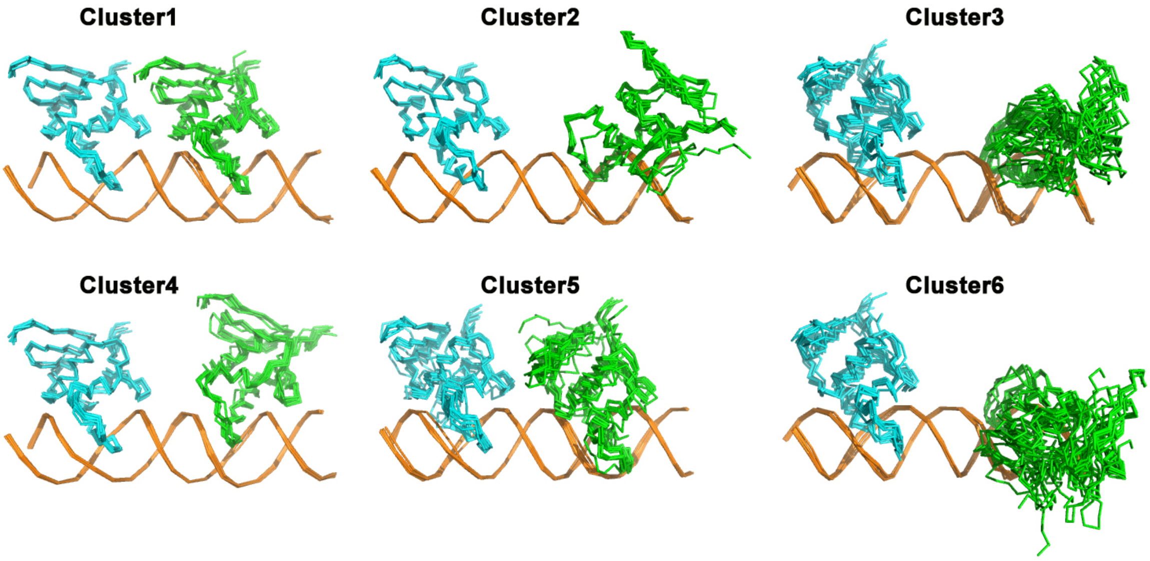

B

Cluster[a]	Haddock score	rmsd[b]	N[c]	E_{vdw}[d]	E_{elec}[d]	E_{AIR}[e]	E_{desolv}[f]	BSA[g]
1	346 ± 8	0.66 ± 0.31	42	131 ± 15	-555 ± 61	1205 ± 43	5.6± 7.2	3905 ±103
2	424 ± 18	2.24 ± 0.75	25	143 ± 16	-382 ± 57	1440 ± 39	13.4± 4.9	3085 ± 134
3	391 ± 23	1.49 ± 0.96	21	153 ± 20	-482 ± 50	1224 ± 87	11.5± 9.5	3144 ± 166
4	429 ± 11	1.55 ± 0.52	18	149 ± 4	-412 ± 39	1456 ± 41	16.9± 6.1	3230 ± 107
5	402 ± 14	1.77 ± 0.89	15	145 ± 21	-470 ± 79	1372 ± 84	14.4± 5.7	3617 ± 93
6	422 ± 12	2.10 ± 1.35	11	185 ± 30	-425 ± 56	1147 ± 96	7.5± 9.8	3150 ± 234

[a] The final 200 structures were clustered based on the pair-wise RMSD matrix using a 3.5 Å cutoff. The statistics are for the 10 lowest energy structures.
[b] Overall backbone RMSD.
[c] Number of structures in a given cluster.
[d] Intermolecular van der Waals and electrostatic energies (kcal mol^{-1}) were calculated with the OPLS parameters using a 8.5 Å cut-off.
[e] HADDOCK ambiguous interaction restraint energy (kcal mol^{-1}).
[f] The desolvation energy (kcal mol^{-1}).
[g] Buried surface area (Å^2).

Supplementary Figure S4. The final 6 clusters of HADDOCK models. (A) The top 10 structures from 6 clusters. All DNA structures are aligned to compare the orientations of 2 $PmrA_C$ molecules. The $PmrA_C$ in half1 site is colored in cyan, in half2 site in green and DNA in orange. (B) Statistics of the top 10 structures from 6 clusters shown in (A).

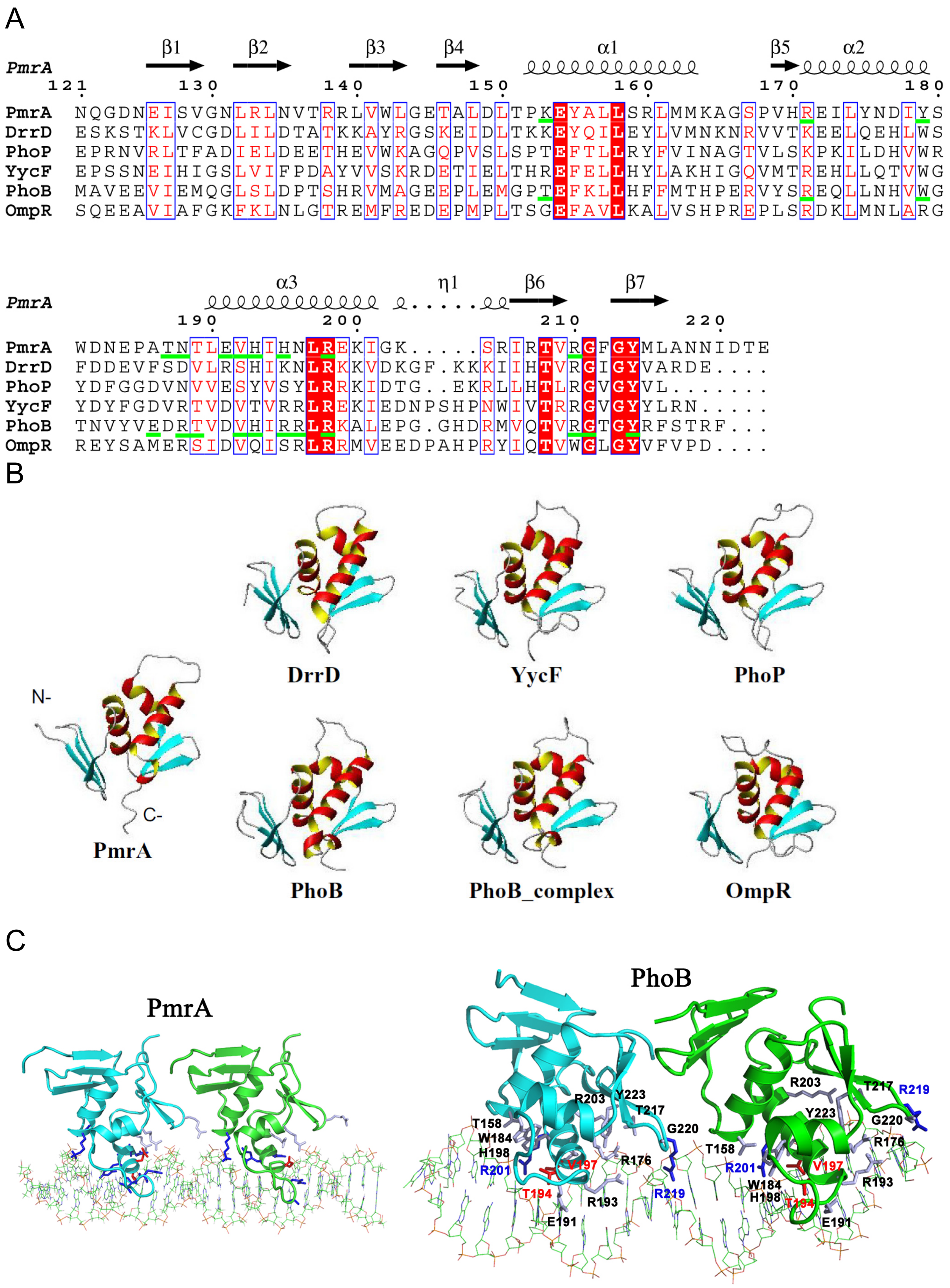

Supplementary Figure S5. (A) Sequence alignment of the effector/DNA-binding domains from response regulator proteins. The residue numbers and secondary structures of *K. pneumoniae* PmrA$_C$ are shown on top. The DNA-contacting residues identified in PmrA$_C$-DNA HADDOCK model and in PhoB$_C$-DNA crystal structure are highlighted with

green underlines. The proteins are *K. pneumoniae* PmrA, *T. maritime* DrrD (PDB code: 1KGS), *B.subtilis* YycF (2D1V), *M. tuberculosis* PhoP (2PMU), *E. coli* PhoB (1GXQ), and *E. coli* OmpR (1OPC). (B) 3D structures of the effector/DNA-binding domains shown in sequence alignment. The structure labeled PhoB_complex is from PDB 1GXP. (C) Structural comparison between $PmrA_C$-DNA HADDOCK model and $PhoB_C$-DNA crystal structure (PDB: 1GXP). The proteins bound to half1 site are in cyan and half2 site green. The residues showing specific H-bond and van der Waals contacts with DNA are in blue and red, respectively. The residues interacting with DNA phosphate backbone are in light-blue.
